# Law and medical practice: A comparative vignette survey of cardiologists in Norway and Denmark

**DOI:** 10.1177/2050312120946215

**Published:** 2020-09-02

**Authors:** Afsaneh Bjorvatn, Anne-Mette Magnussen, Lisa Wallander

**Affiliations:** 1 Western Norway University of Applied Sciences, Bergen, Norway; 2 Lund University, Lund, Sweden

**Keywords:** Hospital treatment, legal regulation, prioritization, medical discretion, case vignettes, Scandinavia, waiting time

## Abstract

**Objective:**

This article explores the implications of legal regulation for medical discretion and decision-making in Norway and Denmark.

**Methods:**

The article is based on a cross-national cross-sectional survey exploring cardiologists’ assessments of patient eligibility for specialist health care. Forty-two cardiologists in Norway and 48 in Denmark were presented with two standardized case vignettes in the form of patient referrals and were asked to assess whether the patient was eligible for treatment by a specialist, and if so, what waiting time would be assigned to the patient.

**Results:**

Primarily based on descriptive statistics, our findings indicate interesting similarities and variations. While there was only minor variation across the countries in cardiologists’ professional assessments about a patient with a more severe condition, judgements of eligibility for specialist treatment varied for a patient with a less severe medical condition. Moreover, Danish cardiologists distinguished between the more severe and less severe conditions to a much lesser extent when assessing eligibility for specialist treatment. For waiting times, there was considerable variation at the general level, from 1 week to 6 months. The assigned waiting times were on average double those for Norwegian cardiologists compared with their Danish counterparts. Denmark’s legal standardization of waiting times appears to lead to shorter waiting times than those prescribed by Norway’s legal regulations.

**Conclusion:**

For a single clear overall intention with a new policy, simpler legal regulations may be more effective than very detailed and specific requirements. If policymakers’ overall intention is for medical doctors to make complex decisions involving the prioritization of patients, then more individualized regulations seem to be a better tool.

## Introduction

Distribution of health care services is a major challenge, and one of the most pressing issues of modern welfare states.^1,2^ As members of the Nordic family of welfare states, both Norway and Denmark are characterized by universal access to health care. At the same time, both countries struggle with increasing health care expenditure, waiting lists and the need to prioritize health care resources in light of new and expensive treatment options. Major legal reforms have taken place in both countries to address these challenges.^3^ In the scholarly literature, these reforms are conceptualized as processes of juridification.^4^ Juridification processes are those involving shifts towards constitutive regulation, increased judicial power, more autonomous judicial conflicts and increased framing of problems in legal and rights-oriented terms.^5^ The main ambition of such legal reforms is to promote and protect social goals such as patients’ equal access to health care services.^6–9^ However, at the general level, the introduction of standardization and regulation in health care entails both benefits and drawbacks for stakeholders. Thus, more precise standards and regulations create more transparency, both for health care providers and for patients. Among other implications, this means that patients obtain a more solid basis for potential individual legal claims.^9^ At the same time, increased standardization may make it more difficult for health care providers to consider a patient’s individual needs.^9^

The current scholarly literature on the regulation of health care distribution and the prioritization of health care resources is dominated by normative studies of the values, norms and principles that should inform decisions on health prioritization, and of prescriptive studies seeking to identify the specific rules, standards and guidelines that prioritization policies should be based upon.^10–13^ Knowledge based on empirical analyses of these same phenomena is scarce.^9,14^ Thus, there are few empirical studies of the legal and administrative regulations at work, including the interpretations and applications of assorted stakeholders, the complex interplay and tensions between the various instruments and actors, and the overall distributive effects of the regulative frameworks.^14^

This study seeks to make an empirical contribution to this field of study by exploring the implications of legal regulations for medical discretion and decision-making. More precisely, our intention is to explore whether and how cardiologists’ professional judgements about patient eligibility for specialist health care services vary between practising cardiologists in Norway and Denmark. The reason for comparing Norway and Denmark is that their institutional and legal contexts are both similar and dissimilar. As members of the same family of welfare states, Norway and Denmark are characterized by similar welfare arrangements, including universal access to health care.^9,15,16^ At the same time, there are noteworthy differences related to the content of recent legal regulations for the allocation of medical services in the two countries. In effect, this means that the discretionary space^17^ delineating medical decision-making varies between the two countries, and Norwegian and Danish medical doctors practising in the same subspecialty must make and justify their professional judgements in accordance with slightly different standards. Thus, if the relatively recent legal regulations have the intended effects on medical decision-making, two patients with identical symptoms could receive different professional advice and medical treatment, depending on the country in which s/he resides. Although this may not be problematic from a legal and/or political perspective, it is still contrary to the professional requirement for comparative consistency, that is, reproducibility, in clinical judgements.^17^

The main reason for choosing cardiologists as respondents for this study was that cardio-vascular medicine is a subspecialty characterized by a relatively high degree of professional consensus, and this field of medicine was one of the first to establish clinical guidelines.^1^ Thus, to the degree that their practice is grounded in their shared professional knowledge, we could expect small variations in the assessments made by cardiologists in Norway and Denmark. However, if their clinical judgements are to some extent guided by the different national laws and regulations in the two countries of concern, we could also expect variation across countries in clinical judgements. Another reason for choosing this particular medical subspecialty is that cardiac patients are one of the largest patient groups receiving treatment in hospitals. For instance, these patients account for about 15% of all hospital admissions in Norway.^18^

To explore cardiologists’ professional judgements about patient eligibility for specialist health care services, we conducted a vignette survey among cardiologists in Denmark and Norway. As part of the survey, the respondents were asked to read two standardized case vignettes in the form of patient referrals and to assess (1) whether the patient described in the referral was eligible for (i.e. needed) treatment by a hospital specialist, and if so, (2) what waiting time they would assign to the patient. They were also asked to justify their assessments.

This article is organized as follows. In section ‘Background’, we describe the background for the study, comprising a description of the legislative frameworks in Norway and Denmark and a short overview of previous research in the field. Section ‘Methods’ describes the research methods used in the study, and Section ‘Results’ presents the empirical results of descriptive statistical analyses. Sections ‘Discussion’ and ‘Conclusion’ conclude with a discussion and summary of the most important findings from the study.

## Background

We first describe the relevant legislative frameworks in Norway and Denmark, with a focus on the two central concerns of this article: the individual’s right to hospital treatment and the waiting time for such treatment. Here, we draw attention to some similarities and differences in the regulations of the two countries. Then, we provide a short overview of some previous research on the implications of the legal regulation of medical practice.

### Regulation of the right to hospital treatment and waiting times in Norway and Denmark

In Norway, a patient’s right to necessary hospital treatment is both clearly articulated and delimited by legislation. The right to specialized health care in hospitals is regulated by the Patient and User Rights Act (Act No. 63 of 2 July 1999) and is supplemented by the Prioritization Regulation (Regulation of 1 December 2000 No. 1208, pursuant to Section 2-1 seventh paragraph of the Patients’ and Users’ Rights Act): *The patient is entitled to necessary health care from the specialist health care services* (Section 2-1 b, second paragraph, first sentence). In Denmark, the Health Care Act regulates the rights of the patient (Consolidating Act No. 1202 of 14 November 2014): *Persons residing in the country have a right to the health care regulated by the Act.* The regulations in both countries express clear ambitions to provide patients with individual entitlements.^9^ However, the content of the right is not entirely clear in either country. The right to *necessary health care* needs general clarification, and the Danish Act is indeterminate when referring to *health care regulated in this Act* and *hospital care*.^9^ In Norway, the Prioritization Regulation explains individual entitlements to specialist health care in more detail. It explicitly restricts the patient’s right to hospital care based on cost-effectiveness considerations, stating that the individual’s right to necessary health care *only applies if the patient can be expected to benefit from the health care, and the costs are reasonable in relation to the effect of the treatment*. Compared with Norway, a Danish patient’s right to hospital care is more vaguely described in the Act, and the delimitation of the entitlement follows principles primarily articulated in health care policies and administrative principles and instructions, which have a weaker legal status and thus provide the patient with a weaker basis for an individual legal claim. In both countries, the content of individual rights to treatment is highly dependent on professional discretion.

Waiting times are a pressing issue in most welfare states, including the Nordic ones. Both Norway and Denmark have legal regulations on maximum waiting times for patients before necessary treatment is provided. In Denmark, patients referred to hospital care have an absolute right to treatment after 1 month, unless an assessment based on medical discretion recommends earlier treatment. This means that patients are given a maximum waiting time, which is good for the purpose of predictability. The right to hospital treatment within 1 month appears to be a strong right. In contrast, the Norwegian regulation in this area is far more individualized and unpredictable as it relies almost entirely on medical discretion relating to the individual patient: *The specialist health services shall, during the period of assessment, cf. the Patient and User Rights Act section 2-2 first paragraph, set a time limit for when the patient, at the latest, shall receive necessary health care. The time limits shall be determined on the basis of what is considered professionally prudent and justifiable.* However, it is considered justifiable for hospital treatment to start within 12 weeks, which is thus stated as a guideline for the start of investigation and treatment.^19,20^ Regarding the regulation of waiting times, the Danish Health Act seems to provide patients with a more standardized and thereby stronger legal tool owing to the provisions stipulating a maximum waiting time of 1 month, which is supplemented with legally binding administrative regulation.

In summary, it is important to note that the regulations in both countries are highly dependent upon medical discretion for their implementation. While the Norwegian regulations provide rather detailed (although vague and open to medical discretion) cost-effectiveness considerations to determine and delimit individual rights, the Danish regulations are formulated in more general terms and thereby leave greater scope for professional standard setting and individual judgement in accordance with political and administrative guidelines and cost-effectiveness considerations.

The Norwegian and Danish legal regulations concerning access to hospital care have both ‘strong’ and ‘weak’ aspects with regard to the rights of patients relating to their clarity, enforceability and independence from political and budgetary decisions.^9^ However, Norway and Denmark use slightly different legal instruments and regulatory strategies to implement the right to hospital care. In this respect, there is room for flexibility in the Danish model that may be advantageous in situations where it is difficult to standardize the assessment of the required treatment. The Norwegian model helps to create transparency about prioritization and prioritization criteria, which are more opaque in the Danish model. The reverse can be said in relation to waiting times; the Danish regulations are more standardized and thus create transparency for the patient, while the Norwegian regulations depend more on individual assessment, which can be less transparent for the patient.

### A short overview of some previous research

As noted, the health care sectors in both Norway and Denmark are characterized by extensive legal regulations. Despite this, there are few empirical studies of the implications of these legal regulations. Among the few existing studies from Norway is one from 2014 on the impact of individual rights on the distribution of health care services in the fields of orthopaedics and cardiology.^1^ The study showed that the formalization of rights to health care services has not led to consistency in the actions of the medical professionals who are supposed to implement the law. In addition, the findings showed that the prioritization guidelines are used differently; while some professionals reported that the legal regulations affect their referral practices, others reported that they do not, indicating substantial variation in the interpretation and implementation of individual rights in professional practice.^1^ Later studies have suggested that over time, the priority guidelines have reduced the previously identified variation to some extent,^21^ but that there remain large regional differences within and between specialties.^22^ Furthermore, in a survey of Norwegian hospital doctors, Bjorvatn and Nilssen^23^ found that 72% know about the priority guidelines, but only 45% consult the guidelines when they are uncertain about the treatment of a patient. In a survey of hospital doctors, it was found that a combination of good knowledge of the priority guidelines and a positive assessment of the previous revision of the guidelines were crucial for the guidelines to influence doctors’ prioritization practices.^24^ There are also more general studies of medical professionals’ beliefs and values indicating that on the whole, medical doctors do not consider the legal regulations to be negative.^23,25^ Carlsen and Bringedal^26^ found that Norwegian hospital doctors have great confidence in guidelines from the health authorities, and the greatest confidence in guidelines that the Norwegian Medical Association has helped to develop. Magnussen and Brandt^1^ noted that medical doctors want even clearer guidelines.

This short overview highlights that we currently know more about medical doctors’ general perceptions of the legal regulations than about the implications that legal rules may have for medical discretion and decision-making.

## Methods

### Respondents

Multiple strategies were used to recruit respondents belonging to the target group of our study, namely cardiologists. The respondent inclusion criteria comprised a specialization in cardiology, or being a member of a Society of Cardiology *and* having experience of assessing referrals for specialized hospital treatment. In Denmark, the respondents were recruited via the Danish Society of Cardiology, who distributed our survey to its members by email (including two reminders). In total, we received 48 valid responses from Danish specialists. The same recruitment strategy proved unsuccessful in Norway. Therefore, we contacted all hospitals in Norway and requested heads of the cardiology departments to distribute the survey to their colleagues. Despite several reminders to the hospitals, we still obtained only a small number of survey responses from Norwegian cardiologists, so we used all available channels (our own professional networks, web pages with contact information for some of the members of the Norwegian Society of Cardiology, article authors, etc.) to recruit respondents for the study. In total, this recruitment strategy yielded 42 valid responses from Norwegian specialists. Approximately, 14% of the cardiologists in Denmark and 10% of the cardiologists in Norway (as of 2017) were represented in our study. The data were collected between October 2017 and December 2018. All the emails sent out to prospective respondents contained information about the study and about their individual rights as participants in a scientific study, thereby giving the respondents the opportunity to make an informed decision about participating in the study. To ensure the respondents’ anonymity, they received the same web link to access the questionnaire. The data collection procedure was approved by the Norwegian Centre for Research Data in 2017.

The majority of our respondents are male (approximately 70% in Denmark and 80% in Norway), and the average age is 48 years in Denmark and 53 years in Norway. The respondents were recruited from all the health regions in Denmark and Norway, with around half operating in the capital regions of their respective countries. At the general level, our respondents constitute a very experienced group of cardiologists, with an average of approximately 8 years’ experience assessing referrals for hospital treatment for the Danish sample and 13 years for the Norwegian sample. Considering our varying strategies for recruiting respondents, our 90 respondents from Denmark and Norway must be regarded as convenience samples of cardiologists. This means that we must exercise caution when generalizing the results from the study to larger populations of cardiologists.

### A vignette survey

This study – comprising a cross-national cross-sectional survey – was based on a questionnaire distributed among cardiologists in Norway and Denmark that used case vignettes to examine cardiologists’ assessments of patient eligibility for specialist health care services. Case vignettes are descriptions of people or situations, and they can be short and simple or long and complex, and they involve either authentic or hypothetical cases.^27–29^ The use of case vignettes to elicit individuals’ knowledge, beliefs, attitudes, values and norms dates back to the 1950s,^30^ and today vignettes constitute an established method both for studying clinical judgements in professional contexts^31,32^ and for developing such judgements in professional education.^33^ This method of collecting data helps to standardize the stimulus across respondents, thereby generating reliable data that allow for comparisons across individuals and settings.^29^ Moreover, by adding context and detail to the object to be evaluated by the respondents, it approximates the complexity of real-life decision-making.^28^ We employed two case vignettes in the form of patient referrals by a general practitioner for specialist treatment in a hospital.

**Figure 1.**
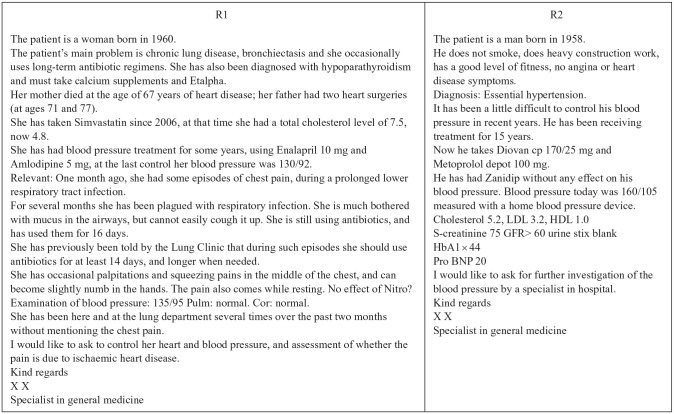
The two referrals (case vignettes) employed in the study.

To develop the case vignettes, we solicited and received a number of randomly chosen and anonymous authentic hospital referrals from two professors of medicine in Norway and Denmark. Our original intention was to use these as a basis for constructing hypothetical referrals to ensure that the general structure and content of the constructed cases would be as genuine as possible. However, after having scrutinized our authentic hospital referrals and realizing that there was indeed great variation in the structure and content of such descriptions (no two referrals were similar), we decided to select two of them for use in the study. To ensure that the contents of the case vignettes were relevant, comprehensible and valid, and that the client conditions described were not so critical (or noncritical) that they would demand an obvious and uniform clinical assessment, the selection of referrals and some minor revisions were conducted in cooperation with three expert physicians from Denmark, Norway and Sweden. Intending to incorporate some real-world variation in the composition of referrals into the study, we deliberately selected one longer and more detailed referral (referral one [R1] and one fairly short referral (referral two [R2]). Figure 1 outlines these two referrals.

The respondents of our study (Norway, n = 42; Denmark, n = 48) subsequently corroborated that the two case vignettes were indeed characterized by high levels of external validity. Thus, R1 was evaluated as ‘realistic’ (i.e. true to a real-life hospital referral) by 83% of the respondents in both Norway and Denmark and as ‘rather realistic’ by 10% of the Norwegian respondents and 15% of the Danish. R2 was evaluated as ‘realistic’ by 64% of the Norwegian respondents and 69% of the Danish respondents. It was considered ‘rather realistic’ by 33% of the Norwegian respondents and 31% of the Danish respondents.

The data for the study were collected using a web questionnaire, which was identical across the two countries except for the language used. The survey was conducted using the online survey software *Questback* (https://www.questback.com/). In the web questionnaire, the case vignettes were displayed one at a time, followed by an identical set of fixed-choice and open-ended questions carefully formulated to measure selected aspects of specialists’ assessments of patient eligibility for hospital treatment (these did not include any formerly validated scales). Most importantly, we asked the respondents to assess (1) whether the patient described in the referral was eligible for (i.e. needed) specialist treatment in hospital, and if so, (2) what waiting time they would assign to the patient. In addition, the respondents were asked to give reasons for their assessments, as well as to evaluate whether their assigned waiting times were short or long. Furthermore, the questionnaire included some more general questions regarding the respondents’ views about quality in hospital referrals (data not included here), and some background questions (see Supplemental material for further details).

### Statistical analyses

In the results section, we present univariate and bivariate statistics related to the respondents’ assessments of (1) whether the patient described in the referral is in need of treatment by a hospital specialist, and if so, (2) what waiting time would be assigned to the patient. We also explore the respondents’ reasons for these judgements, as well as their evaluations of whether their assigned waiting times are short or long. The presentation of results is structured to allow comparisons between referrals (R1 and R2) and, most importantly, between countries (Norway and Denmark). Any conclusions about cross-national differences are based on broad and clearly distinguishable patterns in the data. When large differences across countries are identified, they are accompanied by tests of significance, which allows us to draw conclusions about the robustness of the results.

## Results

To explore and compare cardiologists’ professional assessments of patient eligibility for specialized health care services, we conducted a vignette survey of cardiologists in Denmark and Norway. All respondents read and assessed two case vignettes in the form of patient referrals. R1 contained information about the patient’s gender, age, comorbidities, medical examinations and medications. The referral also included information about the parents’ histories of heart diseases, details of the patient’s current health condition and information about previous consultations. R2 contained information about the patient’s gender, age, general health status, comorbidities, medical examinations and medications. Figure 1 includes the two referrals in full.

### The need for hospital treatment by a specialist

**Figure 2.**
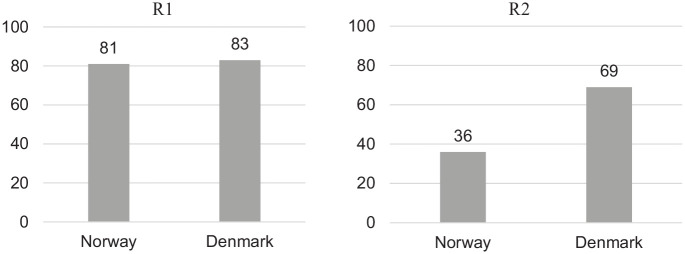
Percentage of respondents who assess that the patient needs treatment by a hospital specialist (Norway, n = 42; Denmark, n = 48).

Figure 2 shows two bar graphs, one for each referral, displaying the percentage of respondents asserting that the patient described in the referral needs hospital treatment.

The large majority of respondents in both Norway (81%) and Denmark (83%) agree that the patient in R1 needs specialist hospital treatment. However, there is disagreement regarding the patient in R2, with 69% of the respondents in Denmark maintaining that the patient needs treatment by a specialist, while only 36% of the Norwegian respondents hold that view. This difference between the two countries with regard to R2 is statistically significant (χ^2^, *p* = 0.002).

The respondents who believed that the patients described in the two referrals did *not* need treatment by a hospital specialist were asked to indicate one or several suggested reasons to support their judgement. Table 1 presents the absolute and relative frequencies of each reason provided in the questionnaire (please note that the analyses are based on a small n).

**Table 1.** Reasons for the assessment that the patient does not need treatment by a hospital specialist (absolute frequencies, percentages in parentheses; multiple responses allowed).

	R1		R2	
	Norway (n = 8)	Denmark (n = 8)	Norway (n = 27)	Denmark (n = 15)
Patient’s need for treatment can be covered by a general practitioner	5 (63%)	7 (88%)	20 (74%)	14 (93%)
Condition is not severe	3 (38%)	0 (0%)	5 (19%)	2 (13%)
Personnel/capacity shortage	1 (13%)	0 (0%)	0 (0%)	0 (0%)
Other	2 (25%)	2 (25%)	10 (37%)	1 (7%)

For both vignettes and across countries, the major reasons for refusing patient referrals for hospital treatment are that the potential treatment can be covered by the patient’s general practitioner and that the patient’s health condition is not considered severe. For the patient in R1, some of the respondents in Norway reported that the lack of diagnostic tests and patient information in the referral were the reasons for not offering hospital treatment to this patient. Several of the respondents who checked the ‘other’ category added in their own words that the patient’s diagnosis in R2 (hypertension) should be managed by a general practitioner or by a nephrologist.

### Assigning waiting times for specialist hospital treatment

**Figure 3.**
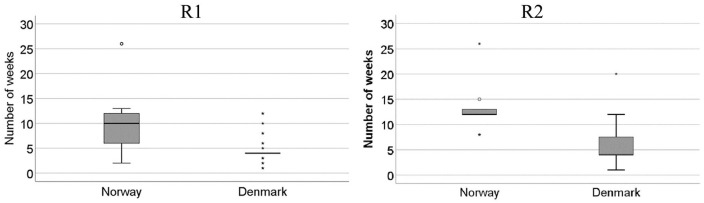
Box plots displaying descriptive statistics for the recommended waiting times (in weeks) for hospital treatment (R1: Norway, n = 34, Denmark, n = 37; R2: Norway, n = 15, Denmark, n = 32).

All the respondents who assessed the patients described in the two referrals as needing treatment by a hospital specialist were asked to assign a waiting time to the patient. A comparison between the average waiting times assigned by cardiologists in Norway and those in Denmark reveals both consensus and variability in responses. If we compare the assigned waiting times across referrals, both the Norwegian and Danish respondents on average assigned longer waiting times for the patient described in R2 (Norway: mean for R1 = 10.3, mean for R2 = 12.8; Denmark: mean for R1 = 4.6, mean for R2 = 6.0). However, comparing the waiting times for each of the two referrals across countries, we see substantial variability, with on average double the waiting time in Norway compared with Denmark. The differences across countries are statistically significant (*t* test, *p* < 0.001). The box plots in Figure 3, one for each referral, illustrate the variability of waiting times in each country.

A box plot splits a ranked distribution of data into quartiles, that is, four groups of equal size. The divisions between the groups are primarily illustrated by the mark in the middle of the box (the median) and the top and the bottom of the box (the upper and lower quartiles). The upper and lower whiskers represent the maximum and minimum values of the distribution, given that these are within a certain range of the upper and lower quartiles (a 1.5 interquartile range). If some observed values are outside these limits, they are treated as outliers and presented as separate dots in the graph. The box plot is a useful way of comparing the variability of assigned waiting times across Norway and Denmark. While a flat box indicates high levels of consensus among the respondents within a country, larger boxes indicate larger variability in the responses. For R1, the median waiting times were 10 weeks (Norway) and 4 weeks (Denmark), and the sizes of the boxes show fairly wide variability in the waiting times assigned by the Norwegian respondents, as against a high level of consensus among the Danish respondents. Indeed, the majority (23/37) of Danish cardiologists assigned a waiting time of 4 weeks to the patient described in R1. For R2, the box plot shows the opposite pattern, with greater variability in the waiting times assigned by the Danish respondents (median = 4 weeks), and a fairly high level of consensus among the Norwegian respondents (median = 12 weeks). Roughly half (8/15) of Norwegian cardiologists assigned a waiting time of 8 weeks. The mode values (not shown) were identical across the two referrals: 12 weeks for Norway (R1 = 11/34 responses; R2 = 8/15 responses) and 4 weeks for Denmark (R1 = 23/37 responses; R2 = 18/32 responses).

After they had assigned a waiting time for specialist hospital treatment, the respondents were asked whether they considered this waiting time to be short or long. In both countries, and for both referrals, approximately half of the respondents assessed their assigned waiting time as short, while the other half considered it to be long. A closer look at the relationships between the assigned waiting times and the evaluations of waiting time length mostly shows positive correlations of medium strength (Pearson’s r > 0.39; *p* < 0.025), indicating that the longer the assigned waiting time, the more inclined the respondent generally is to evaluate it as long. An exception to this clear pattern was found in the Norwegian respondents’ assessments of R2 (Pearson’s r = 0.14). This might be attributable to the low number of respondents in the analysis (n = 15), combined with the small variation in the waiting times assigned by this group. Curiously, the waiting times evaluated as *short* by the Norwegian respondents are on average longer (mean for R1 = 7.1 weeks; mean for R2 = 12.3 weeks) than those evaluated as *long* by the Danish respondents (mean for R1 = 6.0 weeks; mean for R2 = 7.4 weeks).

The respondents were then asked to indicate one or several reasons for their assigned waiting times. Table 2 presents the absolute and the relative frequencies of each of the reasons provided in the questionnaire, and indicates that the rank order is identical across referrals and countries.

**Table 2.** Reasons for the assigned waiting times (absolute frequencies, percentages in parentheses; multiple responses allowed).

	R1		R2	
	Norway (n = 34)	Denmark (n = 40)	Norway (n = 15)	Denmark (n = 33)
Condition is less severe	17 (50%)	22 (55%)	10 (67%)	22 (67%)
Compliance with regulations	12 (35%)	18 (45%)	8 (53%)	13 (39%)
Personnel/capacity shortage	7 (21%)	8 (20%)	2 (13%)	10 (30%)
Condition is severe	6 (18%)	6 (15%)	2 (13%)	0 (0%)
Other	5 (15%)	1 (3%)	0 (0%)	1 (3%)

Thus, while the most common reason for assigning a particular waiting time is that the patient’s health condition is not considered so severe, the second-ranked reason is that the assigned waiting time complies with the legal regulation. Lack of personnel and shortage of capacity were cited as a third reason, most notably by the Danish cardiologists assessing R2. Very few respondents supported their choice of waiting time by citing the severity of the patient’s condition, and the majority of these relate to R1.

## Discussion

At the general level, this study explored the implications of legal regulation for medical discretion and decision-making in Norway and Denmark. As noted, these two countries are members of the same family of welfare states, but use slightly different legal instruments and regulatory strategies to regulate access to health care and thus uphold the right to hospital care. In both countries, the content of individual rights to treatment is highly dependent on professional discretion, although the Norwegian regulations explain individual entitlements to specialist health care in more detail, while in Denmark the right to hospital care is more vaguely described in the Act. Both Norway and Denmark have legal regulations concerning maximum waiting times for patients before necessary treatment is given. In Denmark, patients who are granted the right to hospital care have an absolute right to treatment after 1 month, unless an assessment based on medical discretion recommends earlier treatment. In Norway, the individual rights approach is taken further in the regulations, for example, in the object clause and concerning the limits of patients’ rights and waiting times, according to which each patient is entitled to an individual assessment of the acceptable waiting time.^9^ That said, it is considered justifiable for investigations and treatment to commence within 12 weeks.^19,20^

This article is based on a cross-national cross-sectional survey exploring whether and how cardiologists’ professional assessments about patient eligibility for specialist health care services vary between those practising in Norway (n = 42) and in Denmark (n = 48). Respondents were presented with two standardized case vignettes in the form of patient referrals and asked to assess (1) whether the patient was eligible for (i.e. needed) treatment by a hospital specialist and if so, (2) what waiting time they would assign. The respondents were also asked to justify some of their judgements.

Our findings indicate interesting similarities and variations across Norway and Denmark. First, concerning cardiologists’ assessments of eligibility for specialist treatment in hospital, the majority in both countries found that the patient described in R1 was eligible for such treatment. However, for the second referral, the picture was quite different. While roughly two-thirds of Danish respondents found that the patient in R2 needed to consult a cardiologist, only around one-third of Norwegian doctors were of the same opinion. That all respondents were provided with exactly the same information about the patient’s condition raises the question concerning what can explain such variation. If we consider how the respondents used the information provided in the referrals, the responses to a follow-up open-ended question (data not analysed here) suggest that the Danish and Norwegian medical doctors tended to emphasize (i.e. heed) the same vignette information. This indicates that the medical evaluations of the information stressed in the referrals were similar for the first referral, which describes a patient with a more severe condition, but different for the second one, in which the patient’s condition is less severe. Furthermore, if we consider the rankings of reasons for assessing the patient as *not* eligible for hospital treatment, these were also similar across countries. Thus, both Danish and the Norwegian cardiologists primarily justified their assessments of noneligibility by stating that the patient’s need for treatment could be covered by a general practitioner. One general conclusion from the above results is that while there is only minor variation across countries in medical doctors’ professional judgements about patients with severe conditions, assessments associated with eligibility for treatment by a specialist can vary when the patient’s medical condition is less severe. If we compare the resulting patterns of assessments within countries, while the Danish doctors distinguished between the more severe and less severe conditions to a much lesser extent when making judgements about eligibility for specialist treatment, the opposite was the case for the Norwegian doctors, who largely chose not to prioritize the patient with the less severe condition. In this context, the concept of prioritization entails that some patients are given the right to hospital care while others do not receive the same right.^20^ One possible explanation for the variation in assessments is that the Danish regulation is more open-ended regarding the specific conditions for who has a right to hospital treatment, leaving more scope for medical discretion. In Norway, the level of juridification regarding access and distribution of hospital services is higher, with relatively specific priority guidelines having been introduced to support medical doctors making decisions about whether a patient is entitled to specialist health care services.

Second, concerning the waiting times suggested for patients found to be eligible for hospital treatment, we note extensive variation at the general level, from 1 week to 6 months, with large variations across Norway and Denmark. In both countries, and not surprisingly, the average suggested waiting time was longer for the patient with the less severe medical condition (R2) than for the patient with the more severe condition (R1). Nevertheless, for both referrals, the average assigned waiting times were double those in Norway compared with Denmark. Interestingly, the most frequently suggested waiting time was the same for both referrals within each country: 4 weeks for the Danish cardiologists and 12 weeks for the Norwegian cardiologists. These observations are consistent both with the Danish regulations, where patients have an absolute right to treatment after 1 month, and with the Norwegian guidelines, according to which a waiting time of 12 weeks is considered justifiable. Notwithstanding these overall differences, the rankings of reasons to justify the assigned waiting time were similar across the two countries, with many cardiologists stressing the patient’s less severe medical condition and the fact that the waiting time complied with legal regulations. In general, our results suggest that the legal standardization of waiting times in Denmark seems to lead to shorter waiting times than the legal regulations in Norway, which are more detailed and which promulgate stronger and more specified individual rights (i.e. each patient is entitled to an individual assessment of an acceptable waiting time). In addition, the cardiologists’ responses to a follow-up open-ended question (data not analysed here) provide further support for the hypothesis that the legal regulations indeed affect the assigned waiting times. In fact, both groups of cardiologists underscored that less severely ill patients could actually wait *longer* than the maximum time limit for hospital treatment, but that as a rule they are assigned shorter waiting times in accordance with the regulations. This indicates the risk of a skewed distribution in the allocation of health care services, with diseases and conditions that can be covered by a waiting rationale being given priority at the expense of diseases and conditions where the need for treatment is more chronic.^9^

As is the case with all social science studies, there are some limitations to the present work, the most important of which concerns the fact that our respondents include two fairly small convenience samples. Thus, caution must be exercised when generalizing the results to larger populations of cardiologists. As previously experienced in research on professional assessments, it is generally a challenge to engage very busy professionals such as medical doctors to participate in survey studies.^34,35^ However, considering that cardio-vascular medicine as a subspecialty is characterized by high levels of consensus in professional practice, there is little reason to believe that the cardiologists who chose not to participate in this study would make very different professional assessments than those who chose to take part. Moreover, we doubt that cardiologists’ motives for not participating in the study differed systematically between the two countries. Bearing in mind the limitations associated with our samples, but also drawing on the high levels of standardization in our vignette survey design, we explored the data using mainly descriptive statistics and have based our conclusions about cross-national differences on broad and clearly distinguishable patterns in the data.

## Conclusion

The empirical findings from our study provide support for the contention that regulations matter, but they also give us reason to reflect on the various considerations surrounding the implementation of legal regulations. If a new policy has one clear overall intention, a simpler legal regulation may work better than very detailed and specific requirements. For example, our results suggest that if the overall goal is to reduce waiting times for hospital care, the Danish standardized regulation seems to be a better tool than the more individualized Norwegian regulation. Simple standardized rules also strengthen the patient’s legal position because they increase predictability and clarify the basis for potential complaints. However, these rules seem to have the side effect that Danish doctors differentiate less between patients with more severe and less severe medical conditions compared with their Norwegian counterparts. Thus, if the policymakers’ overall intention is for doctors to make complex decisions involving prioritizing patients, then the more individualized Norwegian regulations seem to be a better tool. Then again, the question about *how* to prioritize patients is not uncomplicated. Indeed, bearing in mind that the distribution of health care services is a major challenge, and one of the most pressing issues of modern welfare states, the question about how the national health services should prioritize between and within various patient groups when capacity is limited is crucial. Our study confirms that juridification processes affect the scope of professional discretion and power in the relationship between the welfare apparatus and recipients of welfare services.

## Supplemental Material

2020_Questionnaire_Supplementary_material – Supplemental material for Law and medical practice: A comparative vignette survey of cardiologists in Norway and Denmark Click here for additional data file. Supplemental material, 2020_Questionnaire_Supplementary_material for Law and medical practice: A comparative vignette survey of cardiologists in Norway and Denmark by Afsaneh Bjorvatn, Anne-Mette Magnussen and Lisa Wallander in SAGE Open Medicine

Bio_Wallander – Supplemental material for Law and medical practice: A comparative vignette survey of cardiologists in Norway and Denmark Click here for additional data file. Supplemental material, Bio_Wallander for Law and medical practice: A comparative vignette survey of cardiologists in Norway and Denmark by Afsaneh Bjorvatn, Anne-Mette Magnussen and Lisa Wallander in SAGE Open Medicine

Short_bio_Afsaneh_Bjorvatn – Supplemental material for Law and medical practice: A comparative vignette survey of cardiologists in Norway and Denmark Click here for additional data file. Supplemental material, Short_bio_Afsaneh_Bjorvatn for Law and medical practice: A comparative vignette survey of cardiologists in Norway and Denmark by Afsaneh Bjorvatn, Anne-Mette Magnussen and Lisa Wallander in SAGE Open Medicine

Short_Bio_Anne-Mette_Magnussen – Supplemental material for Law and medical practice: A comparative vignette survey of cardiologists in Norway and Denmark Click here for additional data file. Supplemental material, Short_Bio_Anne-Mette_Magnussen for Law and medical practice: A comparative vignette survey of cardiologists in Norway and Denmark by Afsaneh Bjorvatn, Anne-Mette Magnussen and Lisa Wallander in SAGE Open Medicine
