# Supplementary material for: Law and medical practice: A comparative vignette survey of cardiologists in Norway and Denmark
Source: SAGE Open Med. 2020 Sep 2;8:2050312120946215. doi: 10.1177/2050312120946215 (PMC7476340; doi:10.1177/2050312120946215)
Supplement: 2020_Questionnaire_Supplementary_material – Supplemental material for Law and medical practice: A comparative vignette survey of cardiologists in Norway and Denmark [file 2020_Questionnaire_Supplementary_material.pdf]

Supplementary material_Questionnaire

The vignettes in the questionnaire contained two referrals with identical questions related to the referrals. For simplicity, both vignettes are given in the following figure.

Vignettes (Referral to hospital treatment)

Referral 1	Referral 2
The patient is a woman born in 1960.<br>The patient’s main problem is chronic lung disease, bronchiectasis and she occasionally uses long-term antibiotic regimens. She has also been diagnosed with hypoparathyroidism and must take calcium supplements and Etalpha.<br>Her mother died at the age of 67 years of heart disease; her father had two heart surgeries (at ages 71 and 77).<br>She has taken Simvastatin since 2006, at that time she had a total cholesterol level of 7.5, now 4.8.<br>She has had blood pressure treatment for some years, using Enalapril 10 mg and Amlodipine 5 mg, at the last control her blood pressure was 130/92.<br>Relevant: One month ago, she had some episodes of chest pain, during a prolonged lower respiratory tract infection.<br>For several months she has been plagued with respiratory infection. She is much bothered with mucus in the airways, but cannot easily cough it up. She is still using antibiotics, and has used them for 16 days. She has previously been told by the Lung Clinic that during such episodes she should use antibiotics for at least 14 days, and longer when needed.<br>She has occasional palpitations and squeezing pains in the middle of the chest, and can become slightly numb in the hands. The pain also comes while resting. No effect of Nitro?<br>Examination of blood pressure: 135/95<br>Pulm: normal. Cor: normal.<br>She has been here and at the lung department several times over the past two months without mentioning the chest pain.	The patient is a man born in 1958.<br>He does not smoke, does heavy construction work, has a good level of fitness, no angina or heart disease symptoms.<br>Diagnosis: Essential hypertension.<br>It has been a little difficult to control his blood pressure in recent years. He has been receiving treatment for 15 years.<br>Now he takes Diovan cp 170/25 mg and Metoprolol depot 100 mg.<br>He has had Zanidip without any effect on his blood pressure. Blood pressure today was 160/105 measured with a home blood pressure device. Cholesterol 5.2, LDL 3.2, HDL 1.0<br>S-creatinine 75 GFR> 60 urine stix blank<br>HbA1×44<br>Pro BNP 20<br>I would like to ask for further investigation of the blood pressure by a specialist in hospital.<br>Kind regards<br>X X<br>Specialist in general medicine

I would like to ask to control her heart and blood pressure, and assessment of whether the pain is due to ischaemic heart disease.
Kind regards
X X
Specialist in general medicine

Please answer the following questions:

1. Does the patient, in your opinion, need to meet a cardiologist/specialist for hospital treatment?
    - Yes
    - No
2. If the answer is *No*, what is the reason for this? It is possible to select multiple answer options.
    - The patient's needs can be met by primary healthcare
    - Personnel/capacity shortage
    - The condition is not severe
    - Other, please specify
3. If the answer is *Yes*, what waiting time for treatment/investigation do you give to the patient? Please specify the number of weeks:
4. If the answer is *Yes*, is the waiting time you have set, in your opinion, a short or long waiting time?
    - Short waiting time
    - Long waiting time
5. If the answer is *Yes*, why are you giving this waiting time? It is possible to select multiple answer options.
    - Personnel/capacity shortage
    - The condition is less severe

- The condition is assessed as severe
- It complies with the regulations
- Other, please specify

6. If the answer is *Yes*, what information in the referral do you consider having the greatest weight? Please specify:
7. How realistic do you think this vignette is? With realistic, it is meant that the referral is similar to a typical referral.

   1 (Not realistic) 2 (Rather realistic) 3 (Realistic)
8. Does the vignette contain sufficient information to assess the patient's needs and set a waiting time for treatment/investigation?
   - Yes
   - No

Background questions

1. Age in years:
2. Gender:
   - Male
   - Female
3. Health region (Norway)
   - South East
   - Vest
   - Mid-Norway
   - North

   Health region (Denmark)
   - Sjælland
   - Capital

- South Denmark
- Mid-Jylland
- North Jylland

4. Are you a cardiologist?
   - Yes
   - No
5. Position:
   - Specialist in management position
   - Specialist
   - Doctor in Specialization
   - Not a doctor/specialist. Please specify:
6. Are you, as part of your tasks, assessing referrals to hospital treatment?
   - Yes
   - No
7. Experience in assessment of referrals. Number of years:
8. Comments regarding the survey
