# Supplementary material for: Law and medical practice: A comparative vignette survey of cardiologists in Norway and Denmark
Source: SAGE Open Med. 2020 Sep 2;8:2050312120946215. doi: 10.1177/2050312120946215 (PMC7476340; doi:10.1177/2050312120946215)
Supplement: Bio_Wallander – Supplemental material for Law and medical practice: A comparative vignette survey of cardiologists in Norway and Denmark [file Bio_Wallander.docx]

Lisa Wallander, [lisa.wallander@soch.lu.se](mailto:lisa.wallander@soch.lu.se)

Present position: Senior lecturer in Social Work, Lund University (2016 - )

Past positions: Senior lecturer in Health & Society, Malmö University (2009 – 2015)

Academic Degree: PhD in Sociology (2008, Stockholm University), Associate Professor of Social Work (2017, Lund University)

Latest publication: Wallander, L., & Blomqvist, J. (2019). Fat words or useful concepts? Consensus and variation in conceptions of problematic drinking in contemporary Sweden. Contemporary Drug Problems (Published online), [https://doi.org/10.1177/0091450919852774](https://doi.org/10.1177%2F0091450919852774)

Main research interests: the sociology of professions, professional judgements, knowledge use in social work practice, substance use and misuse, the factorial survey approach, and quantitative and critical literacy in higher education.
