# Supplementary material for: Law and medical practice: A comparative vignette survey of cardiologists in Norway and Denmark
Source: SAGE Open Med. 2020 Sep 2;8:2050312120946215. doi: 10.1177/2050312120946215 (PMC7476340; doi:10.1177/2050312120946215)
Supplement: Short_bio_Afsaneh_Bjorvatn – Supplemental material for Law and medical practice: A comparative vignette survey of cardiologists in Norway and Denmark [file Short_bio_Afsaneh_Bjorvatn.docx]

Law and medical practice: A comparative vignette survey of cardiologists in Norway and Denmark

Biographic sketch for author: Afsaneh Bjorvatn

Afsaneh Bjorvatn holds a PhD in health economics from the University of Bergen. She is a senior researcher at SNF- Centre for Applied Research at NHH and postdoc at the Western University of applied Sciences, and previously a senior advisor at the Western Norway Regional Health Authority. Her research interests focus primarily on healthcare policy evaluations and cost-benefit analysis. She has published several articles on the implications of financial and legal healthcare reforms and regulations. Of particular relevance are studies of the legal regulation and professional discretion in prioritization of specialist health services, and hospital physicians' attitudes towards financial regulations.
