# Supplementary material for: Law and medical practice: A comparative vignette survey of cardiologists in Norway and Denmark
Source: SAGE Open Med. 2020 Sep 2;8:2050312120946215. doi: 10.1177/2050312120946215 (PMC7476340; doi:10.1177/2050312120946215)
Supplement: Short_Bio_Anne-Mette_Magnussen – Supplemental material for Law and medical practice: A comparative vignette survey of cardiologists in Norway and Denmark [file Short_Bio_Anne-Mette_Magnussen.docx]

**Short bio: Anne-Mette Magnussen**

Anne-Mette Magnussen holds a Dr.Polit (19.05.2006) in political science from the Faculty of Social Sciences, Department of Administration and Organization Theory, University of Bergen, Norway. She is Professor at Faculty of Health and Social Sciences, Department of Welfare and Participation, Western Norway University of Applied sciences Norway. Previously an associate professor and lecturer at Department of Health and Social Sciences, Bergen University College. Her research interests comprises topics as juridification, democracy, law and politics, prioritization of health services, professional discretion.

Magnussen, A.M. and Banasiak, A. (2013): Juridification - disrupting the balance between law and politics? *European Law Journal Vol. 19, No.3, pp 325-339.*

Magnussen, A-M and Nilssen, E. (2013): “Juridification and the construction of social citizenship”. *Journal of Law and Society. Vol. 40, No. 2, pp 228-248*

Aasen, H., Gloppen, S., Magnussen A-M., Nilssen, E. (ed.)(2014): *Juridification and Social Citizenship*. London: Edward Elgar

Aasen, H.S., Hartlev, M. and Magnussen, A-M (2015): ‘Right to Hospital Care and Prioritization from a Law and Politics Perspective – The experiences of Norway and Denmark’ *European Journal of Social Security*, Volum 17 (4) pp 409-435

Aasen, H.S., Bringedal, B., Bærøe, K., Magnussen, A.-M., (ed.) 2018. *Prioritization, governance and equal treatment - Challenges in Norwegian health services*. Cappelen Damm Akademisk, Oslo.
